# MuTE: A MATLAB Toolbox to Compare Established and Novel Estimators of the Multivariate Transfer Entropy

**DOI:** 10.1371/journal.pone.0109462

**Published:** 2014-10-14

**Authors:** Alessandro Montalto, Luca Faes, Daniele Marinazzo

**Affiliations:** 1 Data Analysis Department, Ghent University, Ghent, Belgium; 2 BIOtech, Department of Industrial Engineering, University of Trento, and IRCS-PAT FBK, Trento, Italy; University of Adelaide, Australia

## Abstract

A challenge for physiologists and neuroscientists is to map information transfer between components of the systems that they study at different scales, in order to derive important knowledge on structure and function from the analysis of the recorded dynamics. The components of physiological networks often interact in a nonlinear way and through mechanisms which are in general not completely known. It is then safer that the method of choice for analyzing these interactions does not rely on any model or assumption on the nature of the data and their interactions. Transfer entropy has emerged as a powerful tool to quantify directed dynamical interactions. In this paper we compare different approaches to evaluate transfer entropy, some of them already proposed, some novel, and present their implementation in a freeware MATLAB toolbox. Applications to simulated and real data are presented.

## Introduction

Since its first introduction by Schreiber [1] transfer entropy (TE) has been recognized as a powerful tool to detect the transfer of information between joint processes. The most appealing features of TE are that it has a solid foundation in information theory and it naturally detects directional and dynamical information. Moreover, the formulation of TE does not assume any particular model as underlying the interaction between the considered processes, thus making it sensitive to all types of dynamical interactions. The popularity of this tool has grown even more with the recent elucidation of its close connection with the ubiquitous concept of Granger causality [2], which has led to formally bridge information-theoretic and predictive approaches to the evaluation of directional interactions between processes. Given all these advantages, TE has been increasingly used to assess the transfer of information in physiological systems with several applications in neurophysiology [3]–[6]. It is worth noting that speaking of the transfer of information as measured by TE we refer to the “predictive information transfer” intended as the amount of information added by the past (and present) states of a source process to the present state of a target process.

The estimation of TE from time series data which constitute realizations of the investigated physiological processes is complicated by a number of practical issues that need to be addressed and that are contributing to the development of several recipes to compute this measure.

In this study we discuss three different approaches (binning, nearest neighbor, linear) to evaluate the probability distribution function which constitutes the basis for TE in multivariate systems. In turn, each approach has to be paired with the choice of the time series' past values which contribute information to the knowledge of the present state of a given target time series. The first choice is the classical uniform embedding (UE) that considers a fixed amount of past terms for each series; the second approach is quite recent and employs a non-uniform embedding (NUE) [7], [8] iteratively selecting the most informative terms through an optimization criterion.

These recipes, some of them already established, some novel, are accordingly revisited or explained. Then, in order to contribute to the foundation of a common framework for the application of TE, we describe their implementation in a modular MATLAB toolbox. Several examples are presented allowing a critical comparison of UE and NUE approaches for all the three entropy estimators.

The paper is organized as follows. We first provide an overview of TE. We then distinguish between UE and NUE approaches to the representation of the history of the observed processes. We describe in detail the methods used to estimate the probabilities involved in the evaluation of the TE and their implementation in the toolbox. The approaches are then validated on synthetic time series and then tested on real data: the electroencephalogram of an epileptic patient and cardiovascular measurements in healthy subjects.

## Materials and Methods

### Transfer entropy

Let us consider a composite system described by a set of *M* interacting dynamical subsystems and suppose that, within the composite system, we are interested in evaluating the information flow from the source system 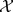 to the destination system 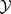, collecting the remaining systems in the vector 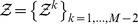. We develop our framework under the assumption of stationarity, which allows to perform estimations replacing ensemble averages with time averages (for non-stationary formulations see, e.g., [9] and references therein). Accordingly, we denote *X*, *Y* and **Z** as the stationary stochastic processes describing the state visited by the systems 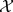, 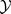 and 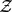 over time, and 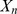, 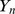 and 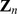 as the stochastic variables obtained by sampling the processes at the present time *n*. Moreover, we denote 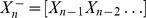, 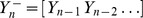, and 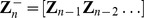 as the vector variables representing the whole past of the processes *X*, *Y* and **Z**. In some cases it can be desirable to take into account also the instantaneous influences of the candidate drivers. In this case, the vectors 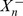 and 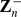 defined above should contain also the present terms 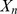 and 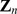. Then, the multivariate transfer entropy from *X* to *Y* conditioned to **Z** is defined as:
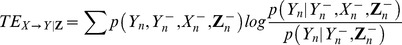
(1)where the sum extends over all the phase space points forming the trajectory of the composite system. 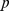
(**a**) is then the probability associated with the vector variable **a** while 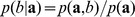 is the probability of observing 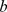 knowing the values of 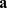. The conditional probabilities used in (1) can be interpreted as transition probabilities, quantifying to which extent the transition of the target system 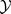 towards its present state is affected by the past states visited by the source system 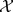. Specifically, the TE quantifies the information provided by the past of the process *X* about the present of the process *Y* that is not already provided by the past of *Y* or any other process included in **Z**.

The formulation presented in (1) is an extension of the original TE measure proposed for pairwise systems [1] to the case of multiple interacting processes. The conditional TE formulation, also denoted as partial TE [5], [8], rules out the information shared between *X* and *Y* that is mediated by their common interaction with **Z**. Note that the TE can be seen as a difference of two conditional entropies (CE), or equivalently as a sum of four Shannon entropies:
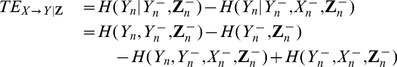
(2)


TE has a great potential in detecting information transfer because it does not assume any particular model that can describe the interactions governing the system dynamics, it is able to discover purely non-linear interactions and to deal with a range of interaction delays [4]. Recent research has proven that TE is equivalent to Granger Causality (GC) for data that can be assumed to be drawn from a Gaussian distribution, a case in which the data covariance is fully described by a linear parametric model [2], [10]. This establishes a convenient joint framework for both measures. Here we evaluate GC in the TE framework and compare this model-based approach with two model-free approaches.

### Reconstruction of the system's past states and TE evaluation

We will discuss here the crucial issue of how to approximate the infinite-dimensional variables representing the past of the processes. This problem can be seen in terms of performing suitable conditioned embedding of the considered set of time series [11].

The main idea is to reconstruct the past of the whole system represented by the processes *X*, *Y*, 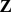 with reference to the present of the destination process *Y*, in order to obtain a vector 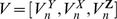 containing the most significant past variables to explain the present of the destination. Once *V* is computed it is easy to evaluate TE as the difference of two CEs or through the four entropies using the whole *V* or convenient subsets of it according to equation (2).

#### Uniform embedding

The large majority of approaches applied so far to estimate TE implicitly follow uniform conditioned embedding schemes where the components to be included in the embedding vectors are selected a priori and separately for each time series. For instance the vector 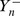 is approximated using the embedding vector 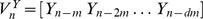, where *d* and *m* are respectively the embedding dimension and embedding delay (the same for 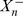 and 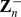, approximated by 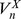 and 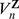). In this way it is possible to distinguish between a first phase during which the past states are collected and a second phase during which the estimate of the entropy, and consequently of the CE, is evaluated by means of the chosen estimator, according to the following pseudo-code:

1. build the vector 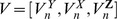;
2. use *V* and 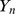 to evaluate the last two entropies of (2) and, consequently, the lowest CE term (CE2);
3. use 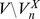 to evaluate the first two entropies of (2) and, consequently, the highest CE term (CE1);
4. compute TE as equal to the difference CE1–CE2.

The obvious arbitrariness and redundancy associated with this strategy are likely to cause problems such as overfitting and detection of false influences [11]. Moreover one should assess which TE values are significant. The significance tests associated with TE estimation based on UE are different for model-based and model free estimators, and are described in the respective following subsections.

#### Non-uniform embedding

Non-uniform embedding constitutes the methodological advance, with respect to the state of art, that we implement as a convenient alternative to UE. This approach is based on the progressive selection, from a set of candidate variables including the past of *X*, *Y*, and 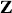 considered up to a maximum lag (*candidate set*), of the lagged variables which are most informative for the target variable 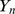. At each step, selection is performed maximizing the amount of information that can be explained about *Y* by observing the variables considered with their specific lag up to the current step. This results in a criterion for maximum relevance and minimum redundancy for candidate selection, so that the resulting embedding vector 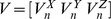 includes only the components of 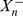, 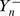 and 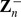, which contribute most to the description of 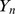. Starting from the full candidate set, the procedure which prunes the less informative terms is described below:

1. Get the matrix with all the candidate terms MC 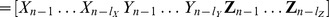, with 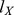, 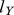, 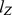 representing the maximum lag considered for the past variables of the observed processes; these matrices will contain also the terms 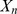 and 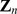 in case one wants to take into account instantaneous effects.
2. Run the procedure to select the most informative past variables and the optimal embedding vector:
  (a) Initialize an empty embedding vector 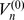

  (b) Perform a while loop on *k*, where *k* can assume values from 1 to the number of initial available candidates, *numC*, in the MC matrix. At the *k-*th iteration, after having chosen 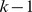 candidates collected in the vector 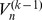:
    for 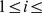 number of current candidate terms
    • add the *i-*th term of MC, 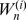, to a copy of 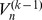 to form the temporary storage variable 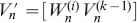

    • compute the mutual information between 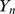 and 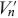, estimating the probability density function according to the chosen estimator
  (c) Among the tested 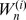, select the term 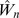 which maximizes the mutual information
  (d) **if** 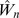 fulfills a test for *candidate significance*, as described below, include it in the embedding vector, 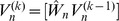, delete it from MC and set 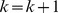.
  (e) **else** end the procedure setting 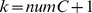 and returning 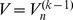

3. Use 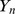 and the full embedding vector 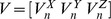 to evaluate the third and fourth entropy values of (2) and, consequently, the lowest CE term (CE2)
4. Take the subset of 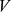 without the past states belonging to the source process, 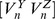 to evaluate the first and the second term of (2) and, consequently, the highest CE term (CE1)
5. compute TE subtracting CE2 from CE1.

As described above, candidate selection is performed maximizing the mutual information between the target variable and the vector of the candidates already selected, incremented by the candidate under examination. As we will see in the following sections, the practical implementation of this general criterion consists of an optimization process (i.e., minimization of the conditional entropy or maximization of the conditional mutual information, depending on the estimator chosen). The performances of the processes mentioned above in the reconstruction of the optimal embedding for an assigned target process are also discussed in [8].

The complexity of the algorithm concerns mainly step 2, in particular step 2(b), involving a *for* loop nested inside a *while* loop: in the worst case the body of the *for* loop is executed 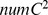 times resulting in a complexity 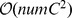.

At step 2(d), the test for candidate significance is performed at the *k*-th step comparing the conditional mutual information between the target variable and the selected candidate given the candidates previously selected up to the 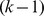-th step, 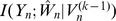, with its null distribution empirically built by means of a proper randomization procedure applied to the points of 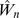. The test for candidate significance is fulfilled if the original measure 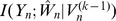 is above the 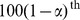 percentile (where 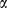 is the desired significance level) of its null distribution. In order to maximize detection accuracy, the adopted randomization procedure is varied for each estimator, and is thus described in the relevant section.

Summarizing, the non-uniform embedding is a feature selection technique selecting, among the available variables describing the past of the observed processes, those who are the most significant - in the sense of predictive information - for the target variable. Moreover, given the fact that the variables are included into the embedding vector only if associated with a statistically significant contribution to the description of the target, the statistical significance of the TE estimated with the NUE approach results simply from the selection of at least one lagged component of the source process. In other words, if at least one component from X is selected by NUE, the estimated TE is strictly positive and can be assumed as statistically significant. If this is not the case, the estimated TE results exactly zero and is assumed as non-significant. This latter case occurs also when the first candidate (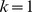) does not reach the desired level of significance, meaning that none of the candidates provides statistically significant information about the target variable. In such a case, that is encountered for instance when the target process is a white noise, the code returns an empty embedding vector and assigns a value of zero to the TE.

### Entropy estimators

Estimation of the TE, performed according to either UE or NUE presented above, results from the application of estimators of entropy and CE to the various terms in (2). The toolbox contains three of such estimators. The first is the linear estimator (LIN) that assumes that data are drawn from a Gaussian distribution. Under this assumption, the two CE terms defining the TE can be quantified by means of linear regressions involving the relevant variables taken from the embedding vector [2]. The second estimator is the classical binning estimator (BIN), which consists of coarse-graining the observed dynamics using Q quantization levels, and then computing entropies by approximating probability distributions with the frequencies of occurrence of the quantized values [12]. The third estimator is based on 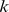-nearest neighbor techniques (NN) which exploit the statistics of distances between neighboring data points in the embedding space to estimate entropy terms; we adopted the bias-reduction method of estimating entropies through neighbor search in the space of higher dimension and range searches in the subspaces of lower dimension [13].

A problem that can arise dealing with UE and NUE procedures when we use entropy estimators that does not assume any probability distribution concerns the curse of dimensionality. Indeed the more candidates we work with, the more the data points will be spread in the phase space, the more the probability density function will assume a constant value. Consequently the NUE should be the most apt method to avoid the curse of dimensionality because it reduces the dimension of the phase space. We will prove this statement in the Results section when it will be clear from the comparison between UE and NUE for the BIN and NN estimators in multidimensional spaces. We are now going to introduce each estimator in detail.

#### Linear estimator (LIN)

The linear estimator method works under the assumption that the overall process 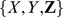 has a joint Gaussian distribution. This assumption allows to work with well-known expressions for the probability density functions. Under this assumption, the two CE terms defining the TE in (2) are expressed by means of linear regressions involving the past states of the systems collected in the vector variables [14]. When the UE is implemented, 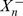 is approximated with the vector of length 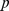, 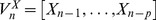, and the same for 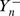 and 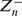 which are approximated by 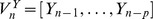 and 
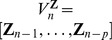
 (here 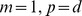). When the NUE is implemented, the embedding vectors will contain only the components resulting from the selection procedure. Then, an unrestricted regression of 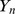 on the full vector 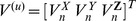, and a restricted regression of 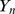 on the reduced vector 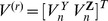, are performed as follows: 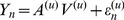
(3)

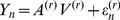
(4)where 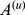 and 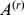 are vectors of linear regression coefficients. The terms 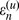 and 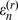 are scalar white noise residuals with variance 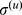 and 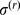. Under the joint Gaussian assumption, it has been demonstrated [2] that the entropy of 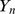 conditioned to the unrestricted or restricted regression vectors is, respectively, 
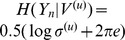
 and 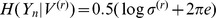, from which follows immediately that:



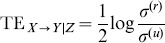
(5)In this study, the unrestricted and restricted regression models in (3) and (4) were estimated by the least-squares method. In the UE implementation, the order *p* of the regressions was selected by the Bayesian information criterion [15]; in the NUE implementation, the order resulted implicitly from the selection procedure. In NUE, maximization of the mutual information between the component 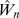 selected at the step *k* and the target variable 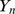 (step 2d) was obtained in terms of minimization of the CE 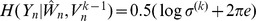, where 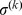 denotes the variance of the residuals of the linear regression of 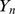 on 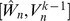. Here, the randomization procedure applied to test candidate significance consisted time-shifting the points of 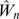 by a randomly selected lag (of at least 20 lags, set to avoid autocorrelation effects) [16].

The statistical significance of the TE estimated through the UE approach is assessed by a parametric F-test for the null hypothesis that the *p* coefficients of 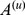 which weigh the past components of the driving process, collected in 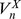, are all zero [17]. In this case, the test statistic is 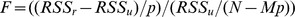, where 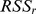 and 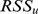 are the residual sum of squares of the restricted and the unrestricted model, and *N* is the time series length. The TE is considered statistically significant if *F* is larger than the value of the Fisher distribution with 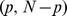 degrees of freedom at the significance level 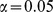.

#### Binning estimator (BIN)

Here we describe the estimator based on fixed state space partitioning. This approach consists of an uniform quantization of the time series followed by estimation of the entropy approximating probabilities with the frequency of visitation of the quantized states [12]. This is the classical approach adopted in the first definition of TE [1]. A time series *y*, realization of the generic process *Y*, is first normalized to have zero mean and unit variance, and then coarse grained spreading its dynamics over 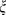 quantization levels of amplitude 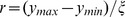, where 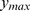 and 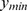 represent minimum and maximum values of the normalized series. Quantization assigns to each sample the number of the level to which it belongs, so that the quantized time series 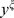 takes values within the alphabet 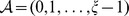. Uniform quantization of embedding vectors of dimension *d* results in an uniform partition of the *d*-dimensional state space into 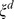 disjoint hypercubes of size *r*, such that all vectors *V* falling within the same hypercube are associated with the same quantized vector 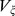, and are thus indistinguishable within the tolerance *r*. The entropy is then estimated as:
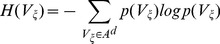
(6)where the sum is extended over all vectors found in the available realization of the quantized series, and the probabilities 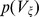 are estimated for each hypercube simply as the fraction of quantized vectors 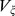 falling into the hypercube (i.e., the frequency of occurrence of 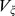 within 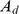). According to this approach, the estimate of TE based on binning results from the application of (6) to the four embedding vectors defined in (2) and determined either by UE or by NUE.

In the NUE implementation, maximization of the mutual information between the component 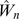 selected at the step *k* and the target variable 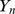 (step 2d) was obtained in terms of minimization of the CE 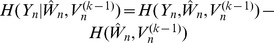, with the two entropy terms estimated through the application of (6). As for the LIN estimator, the randomization procedure applied to test candidate significance consisted in time-shifting the points of 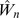 by a randomly selected lag [16].

The statistical significance of the TE estimated through the BIN UE approach exploited the method of surrogate data implemented by the time-shift procedure proposed in [11], [16], [18]. Specifically, the estimated TE is tested against its null distribution formed by the values of TE computed on replications of the original series, where in each replication the source series is time-shifted by a randomly selected lag, set to exclude autocorrelation effects.

#### Nearest Neighbor estimator (NN)

Since its first introduction in 1967 [19], the nearest neighbor method has been shown to be a powerful non-parametric technique for classification, density estimation, and regression estimation. This method can be used to estimate the entropy of a *d*-dimensional random variable *X*, 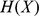, starting from a random sample 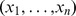 of *N* realizations of *X*. Following the reasoning in [13], if we consider the probability distribution 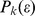 for the distance between 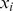 and its *k*-th nearest neighbor, the probability 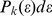 is equal to the chance that there is one point lying within a distance 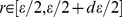 from 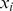, that there are 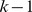 other points at smaller distances from it, and that 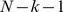 points have larger distances from 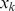. Let 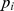 be the mass of the 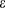-sphere centered at 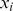, 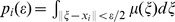, where 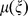 is the density of the variable 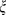. Then, the expectation value of 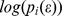 is
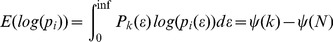
(7)where 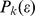 is evaluated through the trinomial formula and 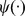 is the digamma function. The expectation is taken here over the positions of all other 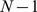 points, with 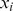 kept fixed. An estimator for 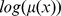 is then obtained by assuming that 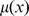 is constant in the entire 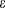-sphere. The latter gives
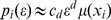
(8)where *d* is the dimension of *x* and 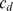 is the volume of the *d*-dimensional unit sphere. For the maximum norm one has simply 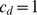, while 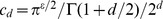 for the Euclidean norm. From (7) and (8) we can evaluate 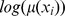 and finally:



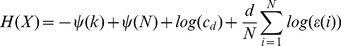
(9)The NN estimator faces the issue of the bias in the estimation of multiple entropies for vector variables of different dimensions by computing entropy sums through a neighbor search in the space of higher dimension, and range searches in the projected sub-spaces of lower dimensions [13]. This approach can be fruitfully exploited for the estimation of the TE, as previously done, e.g., in [3], [4]. To do this, we first rewrite the expression for TE in (2) in terms of the components of the embedding vector 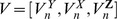 spanning a space of dimension 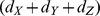:
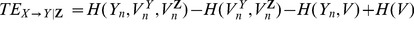
(10)


The term 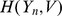 is estimated through neighbor search in the 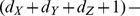
dimensional space, while the three other terms are estimated through range searches in the spaces of dimension 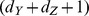, 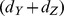 and 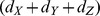. Accordingly, adaptation of (9) to the four terms in (10) yields the equation for TE based on the nearest neighbor estimator:
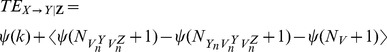
(11)where 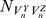,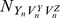 and 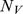 are the number of points whose distance from 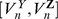, 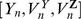 and *V*, respectively, is strictly less than the distance from 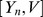 to its *k*-th neighbor, and 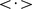 denotes average over all n.

In the NUE implementation of the NN estimator, maximization of the mutual information between the component 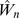 selected at the step *k* and the target variable 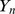 (step 2d) was obtained in terms of maximization of the conditional mutual information 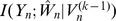, which was computed as described above by estimating the four relevant entropies through a neighbor search in the complete space, and range searches in the projected sub-spaces of lower dimensions. Moreover, the randomization procedure applied to test candidate significance consisted in shuffling randomly and independently both the points of 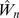 and those of 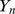. These techniques have been recently shown to be optimal for the selection of candidates in a non-uniform embedding approach using nearest neighbor entropy estimators [8]. As for the BIN UE method, the statistical significance of the TE estimated through the NN UE approach exploited the method of surrogate data implemented by the time-shift procedure proposed in [11], [16], [18].

### Toolbox structure

This section describes how the three TE estimators presented above are implemented in the toolbox, exploiting either the UE or the NUE approach for system state reconstruction.

The same main structure, consisting of the following steps, is common to all methods:

1. normalize the data and perform quantization when needed;
2. evaluate the probability density function (PDF);
3. evaluate CE2 (the second conditional entropy in (2)). This term, accounting for the present state of the target series conditioned to the past of the remaining series including the driver, is evaluated first since it is needed to obtain the complete set of conditional terms including all the series;
4. evaluate CE1 (the first conditional entropy in (2)): this term accounts for the present state of the target series conditioned to a vector including the past of the target series and of the all other series except the driver; such a vector is obtained subtracting the candidates belonging to the driver series from the set of candidates evaluated in the previous step.

Keeping this general scheme in mind, specific steps will be performed for any method of choice. For instance, when using the NUE with the BIN estimator, the steps to be performed are:

1. data quantization;
2. estimation of the PDF, as described in *Binning estimator* section;
3. evaluation of the first and second transfer entropy terms according to *Non-uniform embedding* section.

Given the modularity of the structure shown previously it has been possible to build a user friendly toolbox that allows one to compare all the methods at the same time. The toolbox is available at this link http://dx.doi.org/10.6084/m9.figshare.1005245. The package also contains two existing MATLAB toolboxes which are used in some of the calculations: ARFIT [20], a collection of modules for modeling and analyzing multivariate time series with autoregressive models, used for choosing the model order in LIN UE, and OPENTSTOOL [21], a software package for signal processing with emphasis on nonlinear time-series analysis, and used in searching for neighbor in NN. In order to optimize the toolbox for speed, the routine *evaluateEntropy*, that estimates the entropy among variables according to 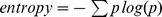, has been converted in a.cpp executable substantially reducing the computation time.

In the following we provide guidelines for the use of the toolbox. Let's start from a hypothetical *main* function and let's explore how a user should set the parameters to chose which methods to use and, possibly, how to build a new method to be inserted within the toolbox.

In the *exampleMain* file, included in the folder /*MuTE/exampleToolbox*, some commented lines remind the method order that has to be kept in mind when setting the parameters, and the parameters available for each method. A first part then follows, devoted to setting the name of the folder that contains data as, for instance, *mat* files. Each file should contain a matrix with the time series as the rows. Then the folders in which all the output files will be stored are defined. In the second part the function *parametersAndMethods* is called.

The function *parametersAndMethods* requires the following inputs, as reported in table 1.

**Table 1:** How to set the input parameters: an example.

Name Parameter	Description
dataDir	folder containing data to be analysed
numProcessors	number of processors used for the parallel session
dataType	filename extension
iresDirGenTS	folder in which results will be stored
dataFileName	data filename
channels	vector containing the series id, among the available series, chosen for the analysis
samplingRate	variable used to resample data
endPoint	value to cut the series length if necessary
autoPairwiseTarDriv	vector containing a 1 or a 0 for each chosen method, reflecting whether TE has to be computed among all the pairs or not. In this latter case, the desired drivers and targets will be specified by *idTargets* and *idDrivers*. By default the instantaneous effects of the drivers are not considered. This can be changed in *parametersAndMethods*, by setting *params_nameMethod.idDrivers = [tarDrivRows(2,:); tarDrivRows(2,:)]*.

- the number of data realizations;
- the sampling rate;
- the subset of interest;
- a value to cut the series length if necessary
- a vector specifying whether each method will take into account all the pairwise combinations of chosen variables. By default the instantaneous effects won't be considered;
- a vector specifying whether the user will set by hand all the pairwise combinations of the chosen variables. This vector will also be used for visualizing the output. It is worth noting that in this case the user should provide as input also the sequence of the destination series and the driver series;
- the folder in which results can be stored, previously defined;
- the folder in which data are stored, previously defined;
- the folder in which results can be eventually copied;
- the number of processors if the code can be run in parallel on several nodes;
- the name of the method chosen and all the relevant parameters as shown in the comments. Here attention should be paid in setting four parameters if the instantaneous effects have to be considered. First of all the function choosing the candidate terms should be set and consequently the variable *usePresent*: *generateConditionalTerm*, and *usePresent* 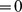 if the instantaneous effects do not have to be taken into account, *generateCondTermLagZero* and *usePresent* 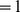 otherwise. Then, if one is interested in the action of more than one driver on a target series, for each driver it can be specified whether its instantaneous effect should be considered by writing twice in a row the number of the driver series. One can also choose which variables belonging to the **Z** set can be considered with their instantaneous effects, filling the vector *idOtherLagZero*, table 2, third column.

**Table 2:** Example of the parameters required to define the methods for an experiment on 5 variables.

	Without Instantaneous Effects	With Instantaneous Effects
Name Parameter	Parameter Value	Parameter Value
genCondTermFun	generateConditionalTerm.m	generateCondTermLagZero.m
usePresent	0	1
idTargets	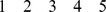	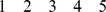
idDrivers	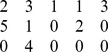	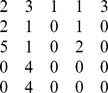
idOtherLagZero	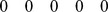	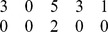

In the second column the instantaneous effects are neglected both for targets and conditioning. In the third column we set instantaneous effects for some drivers and the respective targets. For example, when the target is 1, instantaneous effects are taken into account for driver 2 (first two rows, right column, parameter *idDrivers*) and conditioning variable 3 (first row, right column, parameter *idOtherLagZero*).

For an example of how these parameters should be set, let's consider 5 variables; a conditioned analysis and a vector *idTargets* 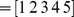 would result in the situation shown in table 2, second column, in which no instantaneous effect are set and the variable *idDrivers* contains on the columns the id of the driver series only once and the variable *idOtherLagZero* is the null vector. An example considering instantaneous effects is reported in table 2, third column, when looking at how drivers 1 and 4 influence the target 2 and how drivers 5 and 2 influence the target 3, with series 5 and 2 as conditoning variables.

The input parameters, including the methods of choice, specified in the function *createNameMethodParams* are stored in a structure called *params* by the function *parametersAndMethods*. This function then computes TE according to the chosen methods, via the function *callingMethods*, and stores the significant results through the function *storingOutput*. In case of multiple realizations/data sets to analyze, the computation can be performed in parallel on separate pools.

The description of the toolbox structure should take into account the structure of the function *callingMethods* that receives in input the data matrix with the time series points in row and the structure *params*. The function reads the names of the methods stored in the *params* structure and computes the TE with all the chosen methods (in parallel if the hardware architecture allows it). This function will return a cell array containing the output of each method.

The open structure of the toolbox allows users to integrate in it their own method. The *main* function should in this case be modified with some comments showing which parameters should be passed as an input to *parametersAndMethods*, and in which order. Each new method should then be implemented following the steps described above using all the necessary parameters conveniently grouped via the function *createNameNewMethodParams*. The new method will be called by setting the appropriate name in *callingMethods*.

The execution time for a single run of the system 14 ranged from 0.4 s for the LIN UE to 90.4 s for NN NUE on a Dell Mini Tower Computer, OptiPlex 990 with four Intel Core i5-2400 CPU at 3.10 GHz, 16 GB of RAM.

One of the purposes of this toolbox is to provide a common framework for all the researchers interested in the application of Transfer Entropy to their data. As part of this effort, MuTE will soon be interfaced with the toolbox TRENTOOL [22]. Readers and users are invited to check periodically the webpages of both toolboxes, that will announce when this interface has been set up.

### Simulated data

The first set of simulated data, implemented to validate the simplest approach to TE, BIN UE, consists of two coupled chaotic maps:
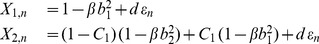
(12)where 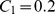 is the coupling coefficient according to which 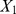 is influencing 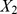, 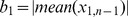, 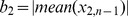, 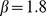, 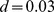 is the coefficient that regulates the noise and 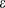 is a Gaussian noise [23]. The function generating these data is *multichaoticmap* available in the folder */MuTE/commonFunctions*.

**Figure 1:**
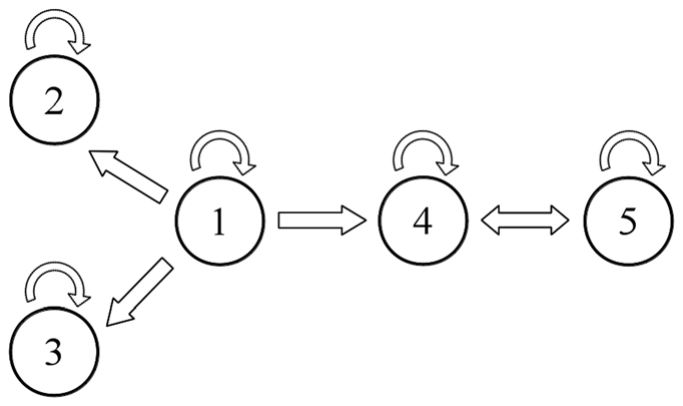
Simulated system. Interactions between the variables of the simulated system.

In the second experiment we simulated five time series in two cases: linear time series, for which we can assume a normal distribution of the variables, and non-linear ones, both generated by an autoregressive (AR) model, equations (13), (14) [24]. The following equations are for the linear Gaussian autoregressive model: 
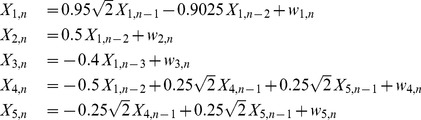
(13)where 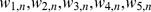 are drawn from Gaussian noise with zero mean and unit variance. The following are the equations for the non-linear model:



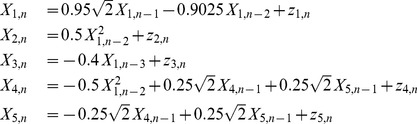
(14)where 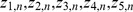 are drawn from Gaussian noise with zero mean and unit variance. A schematic representation of the simulated couplings, valid for both systems, is reproduced in figure 1. The function generating these data is *generateTS* available in the folder */MuTE/commonFunctions*.

### Electroencephalogram in epilepsy

The second experiment is performed on intracranial electroencephalography (EEG) measurements recorded from a patient with refractory epilepsy. The dataset consists of time series from 76 contacts. The first sixty-four of these contacts were placed on a 8×8 grid at the cortical level, while the other 12 were along two six-electrode strips that were implanted in deeper brain structures. Eight sets of measurements were taken on this patient, corresponding to eight different epileptic seizures. An epileptologist, examining the data for each seizure, identified two key periods relating to the seizure i.e., a pre-ictal period, just before the clinical onset, and an ictal one, corresponding to the seizure spread and to the clinical symptoms. Each epoch contained 10 seconds of data recorded at 400 Hz. The data are available at http://math.bu.edu/people/kolaczyk/datasets.html and described in [25]. In order to reduce overfitting, in this application data were downsampled to 100 Hz.

### Cardiovascular and Cardiorespiratory time series

We considered cardiorespiratory time series measured from 15 young healthy subjects (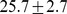 years old) undergoing a standard head-up tilt testing protocol [26]. The acquired signals were the surface electrocardiogram (ECG), the finger arterial blood pressure, and the respiratory nasal flow, measured at 1 kHz sampling rate for 15 minutes in the resting supine position, and 15 further minutes in the 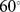 position after passive head-up tilting of the bed table. From these signals, the beat-to-beat variability series of heart period (RR interval), *RR(n)*, systolic arterial pressure (SAP), *Sap(n)*, and respiratory activity, *Resp(n)*, were offline measured respectively as the temporal interval occurring between the *n*-th and the 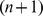-th R waves of the ECG, as the local maximum of the systolic arterial pressure signal inside the *n*-th heartbeat, and as the nasal flow taken at the onset of the *n*-th heartbeat. The time series are available in the folder /*MuTE/cardiovascular_data*. This measurement convention allows instantaneous effects from *Sap(n)* to *RR(n)*, as well as from *Resp(n)* to *Sap(n)* and to *RR(n)*, which were implemented using the relevant feature of the toolbox. The subsequent data analysis was performed on stationary windows of 300 beats taken in supine and upright body positions; inside these windows, the series were normalized to zero mean and unit variance, obtaining the dimensionless series *resp(n)*, *sap(n)*, *rr(n)*.

## Results

### Simulated data

**Figure 2:**
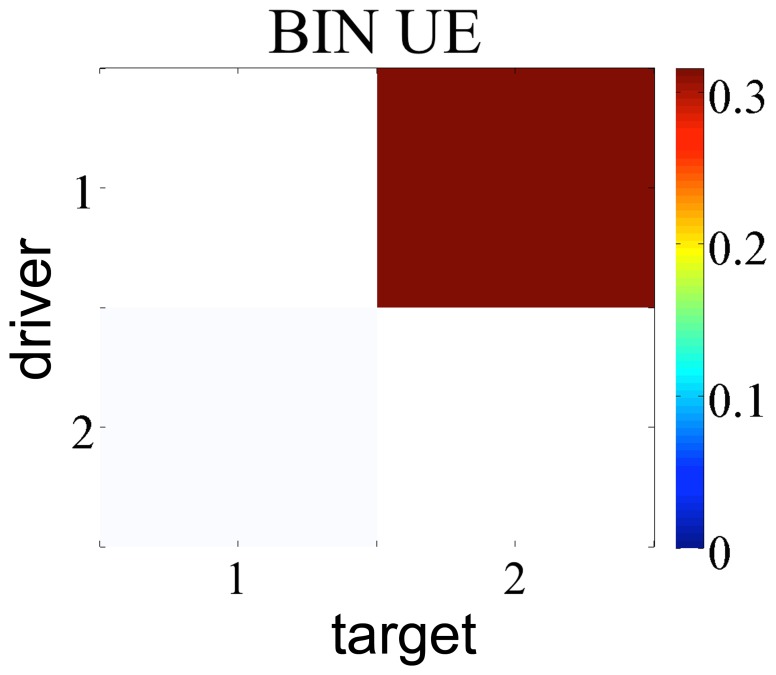
TE matrix representation for the BIN UE estimator applied to the system 12. The color indicates the magnitude of the TE averaged over 100 realizations of the simulation; a shading, inversely proportional to the significance, is superposed to the matrix.

The aim of testing the BIN UE approach on the coupled maps of eq. 12 was to show a simple case of applicability for this method, which constitutes the most basic approach to the model-free evaluation of TE. We generated 100 realizations of eq. 12, each of 512 points, and performed the analysis setting 1 as maximum lag for the candidates, 100 surrogates, 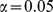 and 6 quantization levels. As we can see in figure 2, the method detected correctly the information transfer returning 100 significant realizations for the link 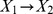 and an average TE much higher than the average TE for the link 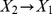 by means of the detection of only 2 significant realizations over 100. We tested also the other methods, which gave similar positive results as the BIN UE, thus demonstrating the applicability of the toolbox for simulations of bivariate systems with short memory.

Then we moved to a more challenging situation in terms of number of interacting systems and lag of the interaction effects, considering the time series simulated with equations 13 and 14, which involve five systems and contain influences up to 3 points in the past. The experiments were run on 100 realizations of eqs. 13 and 14, of length equal to 512 points. We investigated the TE between each pair of variables conditioned to the other three. The setup of the experiment was the following: for all estimators, used either in the UE or in the NUE framework, the maximum lag for the candidates was set as 5, the number of surrogates was fixed to 100 and 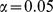. We set 6 quantization levels for BIN and 10 nearest neighbor for NN estimator.

**Figure 3:**
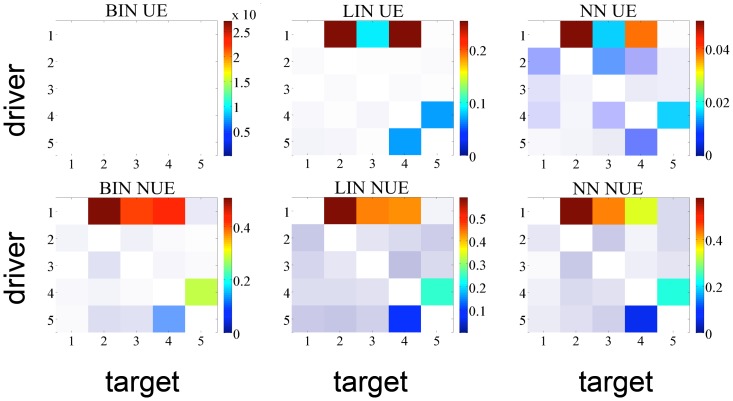
TE matrix representation for all the methods with linear time series of 512 points. The color indicates the magnitude of the TE averaged over 100 realizations of the simulation; a shading, inversely proportional to the significance, is superposed to the matrix.

**Figure 4:**
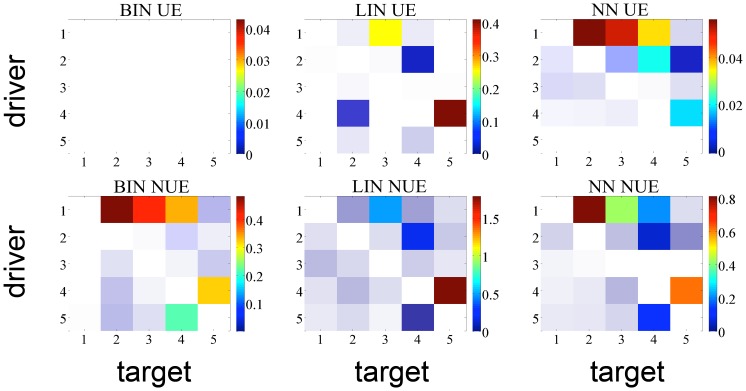
TE matrix representation for all the methods with non-linear time series of 512 points. The color indicates the magnitude of the TE averaged over 100 realizations of the simulation; a shading, inversely proportional to the significance, is superposed to the matrix.

In order to check whether the methods were able to detect the right information transfers, taking into account figure 1, we expect the estimators to find a TE greater than zero with the highest significance at the following matrix elements: (1,2), (1,3), (1,4), (4,5), (5,4). Figures 3 and 4 report the analysis results obtained respectively for the linear system and the non-linear system. Looking at Figure 3 one can notice that LIN UE has very good performances: this reflects the fact that this approach is, in this case of a linear AR system, “by construction”, the most likely to correctly detect information transfer. Its NUE version can detect the same links between the variables, though with a slightly higher number of false positives. The LIN estimator, therefore, is able to reveal the correct information flows for this simulation. On the contrary, BIN UE suffers from the curse of dimensionality mentioned in *Entropy estimators* section: evaluating the influences up to the first 5 past points for all the series implies that the uniform embedding procedure projects the data into a phase space of 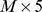 dimensions, where *M* is the number of time series, resulting in a phase space with 25 dimensions, with the points spread enough to lose relevant information about the transfer entropies in the system. As a consequence, no significant link is retrieved with this approach. NN UE retrieves all the true links, but also detects a number of false interactions. Its better performance compared with BIN UE reflects the ability of the nearest neighbor approach to achieve bias compensation in the estimation of entropies of variables of different dimension. Still, the performance of NN UE is not optimal due to the curse of dimensionality. On the other hand, BIN and NN used in the NUE framework are able to recover all the correct links, with only a few false positives. Moving to Figure 4 depicting TE analysis for the non-linear systems, one can notice that the LIN estimator cannot detect all the correct information flows, returning in addition some false positives. Again, BIN UE cannot detect any link because of the curse of dimensionality; conversely BIN NUE, in which the dimensionality of the space is considerably reduced, has high specificity and sensitivity. NN NUE can achieve almost the same performance as BIN NUE but its specificity is lower, especially along the direction 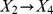. NN UE this time is not able to detect all the correct information transfers (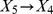 remains undetected) and reveals some false positives (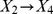, 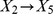).

**Figure 5:**
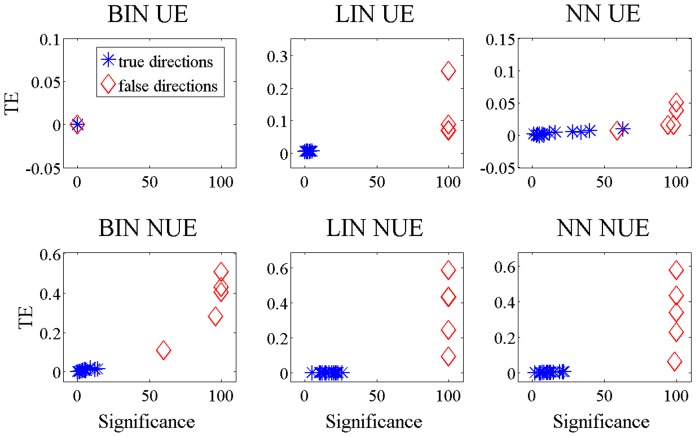
TE values versus the number of significant realizations, linear system. For time series of 512 points simulated according to 13, the links retrieved by the different methods are reported. The five simulated links are red; those who are not present in the model are blue.

**Figure 6:**
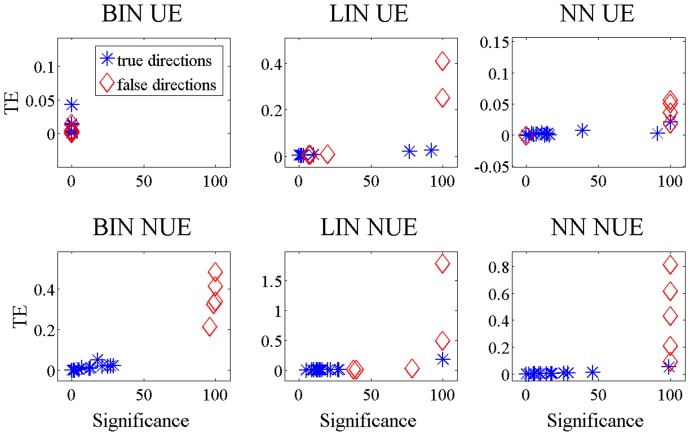
TE values versus the number of significant realizations, non-linear system. For time series of 512 points simulated according to 13, the links retrieved by the different methods are reported. The five simulated links are red; those who are not present in the model are blue.

To better clarify whether and how much the methods are able to distinguish between the true information transfer links and the false ones, in Figures 5 and 6 we plotted the average TE with respect to the number of significant realizations found by the methods. Each retrieved link is a point in this bidimensional space. The true links should be in the upper right corner of the plot corresponding to high TE and high number of significant realizations, and they should be apart from the false links, whose natural location would be around the origin of the plot (low TE and low number of significant links). Looking at figure 5 one can notice that for all the methods, except BIN UE and partly NN UE, the two groups of links are well separated and the false links with an averaged TE greater than zero in figure 3 can be neglected. The opposite reasoning holds for BIN UE that is not able to distinguish between false and true links. For the non-linear system (figure 6) only BIN NUE can separate well true positive from false positive links.

**Figure 7:**
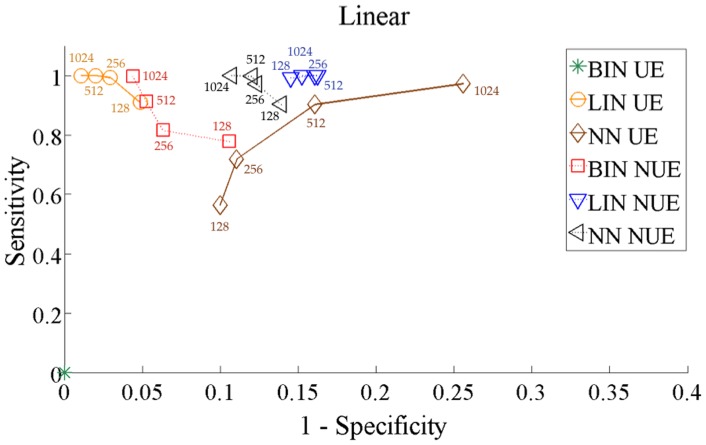
ROC curves for all methods for the linear system. The curves are obtained reporting the results obtained gradually increasing the time series length simulated according to 13 from 128 to 1024 points.

**Figure 8:**
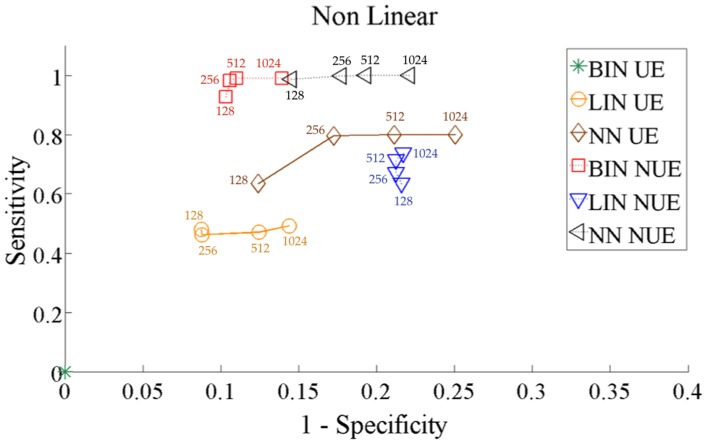
ROC curves for all methods for the non-linear system. The curves are obtained reporting the results obtained gradually increasing the time series length simulated according to 14 from 128 to 1024 points.

To understand how stable the performance of the methods is, in terms of sensitivity and the specificity, with respect to the length of the analyzed data set, we computed the analysis varying the series length from 128 to 1024 points. Figures 7 and 8 depict, respectively for the systems 13 and 14, the Receiver Operating Characteristic (ROC) curves obtained for all methods as a function of the series length. Evaluating the amount of TP (true positives), TN (true negatives), FP (false positives) and FN (false negatives) after grouping together all coupled directions (positives) and all uncoupled directions (negatives), we computed sensitivity as 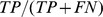 and specificity as 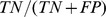. In the case of the linear system (Figure 7), all methods except the BIN UE provide good performance, with the LIN estimator providing the best sensitivity and specificity. All methods provided robust results with respect to the series length, with only a limited decay in the performance observed for 128 points. In the case of the non-linear system (Figure 8), the performance was optimal for BIN NUE and NN NUE (with a slightly lower specificity), while the methods implementing either the LIN estimator or the UE approach were considerably less sensitive.

### Electroencephalogram in epilepsy

**Figure 9:**
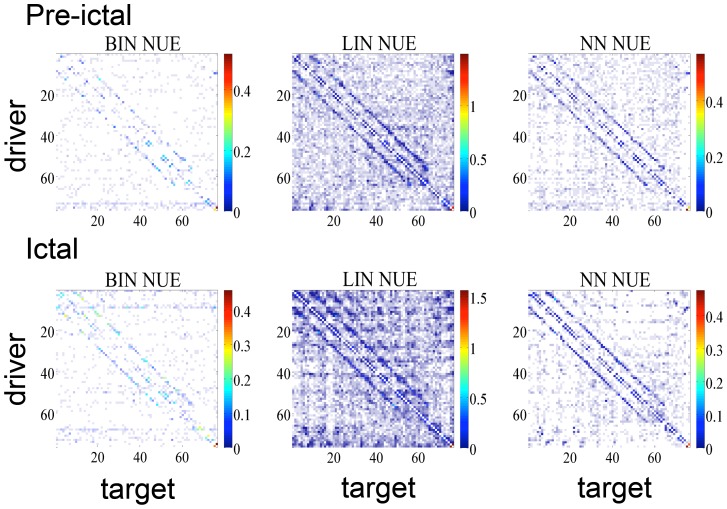
TE matrices for human EEG recordings. Matrices of Transfer Entropy among the 76 intracranial contacts implanted in an epileptic subject. Contacts 1 to 64 belong to a cortical grid, contacts 65 to 76 to two strips implanted in deeper structures. Transfer Entropy values are obtained with three approaches to non-uniform embedding considering ten seconds of brain activity in the pre-ictal phase (top panels) and ictal phase (bottom panels). The color scale reflects Transfer Entropy values, the shading is inversely proportional to the significance: brighter colors correspond to more significant values.

In such high dimensional and redundant data, a non-uniform embedding approach is intuitively the most appropriate to identify the patterns of information transfer specific to the onset and spread of the epileptic seizure. The aim of the experiment was to use the NUE approach in order to characterize the dynamical interactions in the epileptic brain by looking at the information transfer between the variables during the pre-ictal and ictal phases. The embedding size in the embedding matrix (EM) was set to eight. The results are reported in figure 9. The regions corresponding to one of the depth strips (contacts 70 to 76) and the lower left corner of the grid (contacts 1–4, 9–11 and 17) were resected during anterior temporal lobectomy as they were identified by the epileptologists as the seizure onset zone. The Binning approach to NUE seems to be the one which best identifies these areas as those most influential at the start of the seizure and in the early phases of the spread, signature of a putative seizure onset zone. The Binning approach is more selective with respect to the target variables for each driver and less sensitive to the confounding effect of volume conduction resulting in the diagonal patterns observed with the other methods and probably due to conduction effects on the grid.

### Cardiovascular data

**Figure 10:**
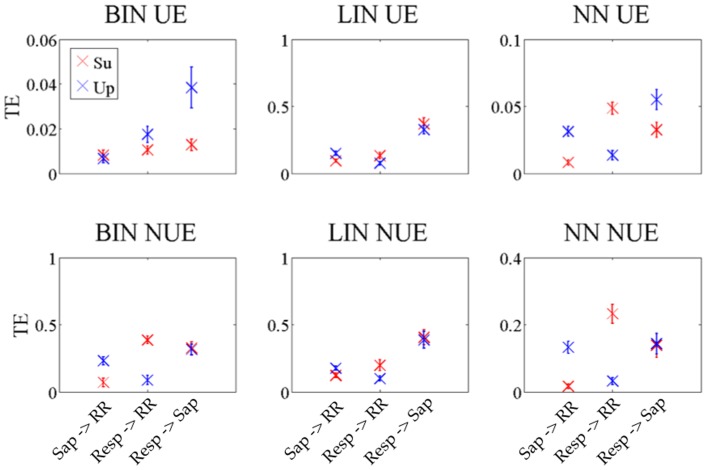
Transfer entropy for the links of interests in the cardiovascular example. In red the TE for the subjects in supine position, in blue the TE for the subjects in upright position. The error bars represent the standard error.

The analysis of the information transfer for cardiovascular and cardiorespiratory time series was focused on the directions of interaction that are more studied from a physiological point of view: the link from SAP to RR which is related to the so-called cardiac baroreflex, and the links originating from Resp and directed either to RR or to SAP, related respectively to cardiopulmonary or vasculo-pulmonary regulation mechanisms [26]. The particular protocol considered allows to establish a sort of verifiable ground truth. Indeed, in the studied protocol, the transition from supine to upright is known to evoke an activation of the sympathetic nervous system and a concurrent deactivation of the parasympathetic nervous system [27]. Accordingly, the two main physiological regulation mechanisms that are expected to be solicited by this transition are: (i) a substantial increase of the baroreflex regulation (direction 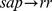), reflecting the necessity of the cardiovascular system to react with changes in the heart rate to the higher fluctuations in the arterial pressure induced by the sympathetic activation; and (ii) a substantial decrease of cardiopulmonary regulation (direction 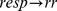), reflecting the dampening of respiratory sinus arrhythmia consequent to the parasymphatetic deactivation [28]. On the contrary, no known alterations of the vasculo-pulmonary regulation (direction 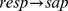) are expected when moving from supine to upright [26]. In our analysis all these trends are well reflected in terms of information transfer when the multivariate TE is estimated using the BIN NUE and NN NUE methods. Figure 10 reports the distribution of the multivariate TE computed along these directions using all methods, with subjects studied in the supine and upright body positions. We observe in Figure 10 that BIN NUE and NN NUE reveal, moving from supine to upright, a substantial increase of the TE from Sap to RR, a substantial decrease of the TE from Resp to RR, and an unchanged TE from resp to SAP. These trends were also observed, though with less evident differences, computing the TE according to the LIN estimator. These results suggest the appropriateness of model free TE estimators based on NUE for detecting the information transfer in physiological time series. On the contrary, the BIN UE estimator shows different trends of difficult physiological interpretation, thus suggesting also in experimental data that the estimated TE may be unreliable due to the curse of dimensionality.

## Conclusions

In this work we have considered three entropy estimators able to reveal the information transferred among variables represented by time series. We implemented the estimators in two different ways according to UE and NUE approaches, resulting in six methods, two of which are novel, BIN NUE and NN NUE. We compared all the methods validating them on simulated data first and then on real data. We checked whether and how the methods were affected by the number of variables and by the time lag at which the series influenced each other. From the results obtained we can conclude that the new methods introduced, not assuming any model to explain the data and exploiting the NUE strategy for component selection, can detect the correct information flows and are less affected by the number of involved processes and by their interaction lags. The NUE approaches are indeed prone to work in high dimensional spaces as well as in low dimensional spaces because of their ability to reduce the effective dimension of the phase space, choosing only the right variables at the specific time lag that are better able to explain the destination series. On the contrary, BIN UE and NN UE suffer from the curse of dimensionality when several time series and longer interaction delays are present. Finally, looking at LIN UE and LIN NUE performances we can conclude that, even though the equivalence between Granger causality and TE establishes a convenient joint framework for these two measures, there are some drawbacks in having a predefined model to explain the data when these are non-linear. The better performances obtained by the new methods appear when looking at the ROC curves: BIN NUE and NN NUE have high sensitivity and specificity both for linear and non-linear systems.

All the methods have been implemented in an organic toolbox in MATLAB, allowing straightforward comparisons between the methods, and flexible enough to allow other users to implement their own methods.

## References

[1] Schreiber T (2000) Measuring information transfer. Phys Rev Lett 85: 461. 10991308 10.1103/PhysRevLett.85.461

[2] Barnett L, Barrett A, Seth A (2009) Granger causality and transfer entropy are equivalent for gaussian variables. Phys Rev Lett 103: 238701. 20366183 10.1103/PhysRevLett.103.238701

[3] Wibral M, Rahm B, Rieder M, Lindner M, Vicente R, et al (2011) Transfer entropy in magnetoencephalographic data: Quantifying information flow in cortical and cerebellar networks. Prog Biophys Mol Biol 105: 80–97. 21115029 10.1016/j.pbiomolbio.2010.11.006

[4] Vicente R, Wibral M, Lindner M, Pipa G (2011) Transfer entropy a model-free measure of effective connectivity for the neurosciences. J Comput Neurosci 30: 45–67. 20706781 10.1007/s10827-010-0262-3 PMC3040354

[5] Vakorin VA, Kovacevic N, McIntosh AR (2010) Exploring transient transfer entropy based on a group-wise ica decomposition of eeg data. Neuroimage 49: 1593–1600. 19698792 10.1016/j.neuroimage.2009.08.027

[6] Gourévitch B, Eggermont JJ (2007) Evaluating information transfer between auditory cortical neurons. J Neurophysiol 97: 2533–2543. 17202243 10.1152/jn.01106.2006

[7] Faes L, Nollo G, Porta A (2011) Information-based detection of nonlinear granger causality in multivariate processes via a nonuniform embedding technique. Phys Rev E 83: 051112. 10.1103/PhysRevE.83.051112 21728495

[8] Kugiumtzis D (2013) Direct-coupling information measure from nonuniform embedding. Phys Rev E 87: 062918. 10.1103/PhysRevE.87.062918 23848759

[9] Ledberg A, Chicharro D (2012) Framework to study dynamic dependencies in networks of interacting processes. Phys Rev E Stat Nonlin Soft Matter Phys 10.1103/PhysRevE.86.041901 23214609

[10] Hlavácková-Schindler K (2011) Equivalence of granger causality and transfer entropy: A generalization. App Math Sci 5: 3637–3648.

[11] Vlachos I, Kugiumtzis D (2010) Nonuniform state-space reconstruction and coupling detection. Phys Rev E 82: 016207. 10.1103/PhysRevE.82.016207 20866707

[12] Hlaváčková-Schindler K, Paluš M, Vejmelka M, Bhattacharya J (2007) Causality detection based on information-theoretic approaches in time series analysis. Phys Rep 441: 1–46.

[13] Kraskov A, Stögbauer H, Grassberger P (2004) Estimating mutual information. Phys Rev E 69: 066138. 10.1103/PhysRevE.69.066138 15244698

[14] Barrett AB, Barnett L, Seth AK (2010) Multivariate granger causality and generalized variance. Phys Rev E 81: 041907. 10.1103/PhysRevE.81.041907 20481753

[15] Schwarz G (1978) Estimating the dimension of a model. Ann Stat 6: 461–464.

[16] Quiroga RQ, Kraskov A, Kreuz T, Grassberger P (2002) Performance of different synchronization measures in real data: a case study on electroencephalographic signals. Phys Rev E 65: 041903. 10.1103/PhysRevE.65.041903 12005869

[17] Brandt P, Williams J (2007) Multiple Time Series Models. Number No. 148 in Multiple Time Series Models. SAGE Publications

[18] Faes L, Porta A, Nollo G (2008) Mutual nonlinear prediction as a tool to evaluate coupling strength and directionality in bivariate time series: comparison among different strategies based on k nearest neighbors. Phys Rev E 78: 026201. 10.1103/PhysRevE.78.026201 18850915

[19] Cover T, Hart P (1967) Nearest neighbor pattern classification. IEEE Trans Inf Theory 13: 21–27.

[20] Schneider T, Neumaier A (2001) Algorithm 808: Arfit: a matlab package for the estimation of parameters and eigenmodes of multivariate autoregressive models. ACM Trans Math Softw 27: 58–65.

[21] Merkwirth C, Parlitz U, Wedekind I, Engster D, Lauterborn W (2009) Opentstool user manual. Göttingen, Germany: Drittes Physikalisches Institut, Universität Göttingen.

[22] Wibral M, Vicente R, Priesemann V, Lindner M (2011) Trentool: an open source toolbox to estimate neural directed interactions with transfer entropy. BMC Neurosci 12: P200. 10.1186/1471-2202-12-119 PMC3287134 22098775

[23] Marinazzo D, Pellicoro M, Stramaglia S (2008) Kernel method for nonlinear granger causality. Phys Rev Lett 100: 144103. 18518037 10.1103/PhysRevLett.100.144103

[24] Baccalá LA, Sameshima K (2001) Partial directed coherence: a new concept in neural structure determination. Biol Cybern 84: 463–474. 11417058 10.1007/PL00007990

[25] Kramer MA, Kolaczyk ED, Kirsch HE (2008) Emergent network topology at seizure onset in humans. Epilepsy Res 79: 173–186. 18359200 10.1016/j.eplepsyres.2008.02.002

[26] Faes L, Nollo G, Porta A (2011) Information domain approach to the investigation of cardio-vascular, cardio-pulmonary and vasculo-pulmonary causal couplings. Front Physiol 2: 80. 22069390 10.3389/fphys.2011.00080 PMC3209583

[27] Cohen MA, Taylor JA (2002) Short-term cardiovascular oscillations in man: measuring and modelling the physiologies. Physiol J 542: 669–683. 10.1113/jphysiol.2002.017483 PMC2290446 12154170

[28] Nollo G, Faes L, Antolini R, Porta A (2009) Assessing causality in normal and impaired short-term cardiovascular regulation via nonlinear prediction methods. Philos Trans A Math Phys Eng Sci 367: 1423–1440. 19324717 10.1098/rsta.2008.0275

